# Ruharo Mission Hospital eye drop production facility

**Published:** 2023-05-22

**Authors:** Dan Kuguminkiriza, Abeer H A Mohamed-Ahmed

**Affiliations:** Pharmacist: Eye Drop Production Unit, Ruharo Eye Centre, Ruharu Mission Hospital, Mbarara, Uganda.; Research Fellow in Pharmacology and Clinical Trials Management: London School of Hygiene & Tropical Medicine, UK.

**After just four years, this hospital-based facility in Uganda has become licenced to produce ten different eye medicines, making them more affordable for patients and easier to obtain.**

**Figure 1:**
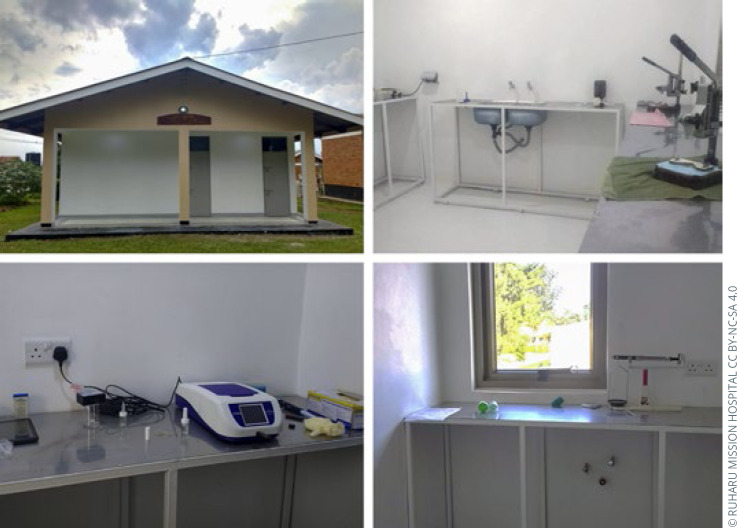
Ruharo Mission Hospital's eye drop production facility. Clockwise from top left: the external building, the clean production room, the quality control room, and the raw material preparation room. Adapted from^1^.

Ruharo Eye Care Centre is part of Ruharo Mission Hospital in Uganda and offers care for patients suffering from a wide range of eye diseases, such as cataract, glaucoma, and corneal infections.

In order to provide high quality eye drops for common eye conditions, at a price people can afford, an eye drop production unit was set up in 2019.

The unit began as a small laboratory that prepared eye drops in small batches, starting with 50 units at a time. A bucket system was used for sterilisation. As demand increased, the unit began producing 100 units per batch and eventually this grew to 400 units per batch.

However, meeting the demand and sourcing ingredients soon became a challenge. The unit decided to apply for a manufacturing permit, which would enable them to import their own raw materials and packaging. During the application process, Uganda's National Drug Authority, which regulates manufacturing permits, inspected the facility and stated that a more sterile working environment would be needed before they could approve a permit.

This required a significant financial investment, of around US $30,000. The eye unit could cover the cost using the funds it had accumulated over the preceding two years. Since the unit was already providing 90% of the eye medicines used in Ruharo Eye Centre at that time, the long-term benefits made this a worthwhile investment.

The eye drop production facility is now fully licenced by the Ugandan National Drug Authority to produce terminally sterilised eye drops. It produces more than ten different eye medicines and diagnostics, including fluorescein, cycloplegics, mydriatics, antibiotics, anti-inflammatories, and specific treatments for glaucoma, microbial keratitis, allergic conjunctivitis, and dry eyes.

Most raw materials are imported from China; cleaning agents are sourced in Uganda, and packaging materials are imported from the UK and China.

The eye drop production facility acts an independent unit under the general management of the hospital. The facility is run by four technical and two non-technical staff members:

- a chemist, who is also the production officer
- a pharmaceutical scientist and a laboratory scientist, who act as quality control officers
- a pharmacist, who is also the supervisor and quality assurance officer.

The cost of production per bottle is approximately US $0.50, and units are released for an average cost of US $1.70 per unit. The revenue generated is either reinvested into the facility or used to support the running costs of the hospital.

The biggest consumer of the locally manufactured eye drops is Ruharo Eye Centre clinic, which uses 60% of the eye drops produced. Other eye hospitals in the region also order from the unit, and steps are being taken to acquire a national distributor.

## The facility

The eye drop production unit consists of a clean room (class C), a sterilisation room, and a quality control room (see Figure 1). The clean room is equipped with an airflow-controlled environment HVAC (heating, ventilation, and air conditioning) system, which includes a 0.4 µm filter capable of removing dust and particulates from the air. The HVAC system provides clean air and temperature control within the facility.

## Equipment and critical processes used in Ruharo eye drops production unit

The equipment used and the process are described below:

### 1. Storage of raw materials

Raw materials are stored in a room in which both temperature and relative humidity are controlled and monitored using a hygrometer/ thermometer digital reader.

### 2. Weighing raw materials

**Figure 2:**
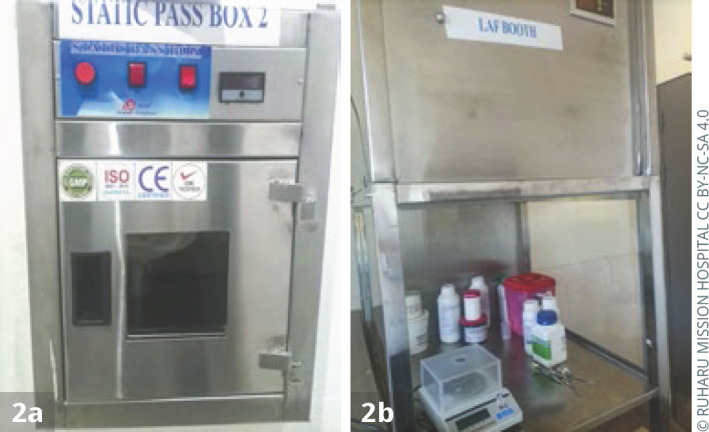
Ruharo Mission Hospital eye drop production facility **a.** Static pass-box for UV sterilisation of items. **b.** Laminar air flow booth to weigh ingredients and materials under aseptic conditions.

- Raw materials are transferred from the storage room to the weighing room passing through a static pass-box with UV lamp (Figure 2). The UV lamp sterilises the raw materials, packaging and powder containers by irradiation.
- The weighing room has an extraction fan which creates negative air pressure in the room, causing the entry of fresh air through the 10-micron filters.
- Weighing is carried out in sterile conditions provided by a laminar air flow booth with HEPA filters (Figure 2), using a digital balance with a range from 0.01 g to 5,000 g.

### 3. Transfer of raw materials from the weighing room to the clean room

Weighed powders and other raw materials are passed through a second pass box (with UV irradiation) to the Class C clean room (Figure 2).

### 4. The clean room

The cleanliness of this room maintained by:

- Cleaning all surfaces, including floor, walls, and ceiling, with antiseptic solution, prepared according to the manufacturer's instructions.
- Spraying all surfaces using 70% ethanol prior to start of production.
- An HVAC system, which maintains the purity of air circulated in the room.

### 5. Mixing and filtration of eye drop solutions

**Figure 3:**
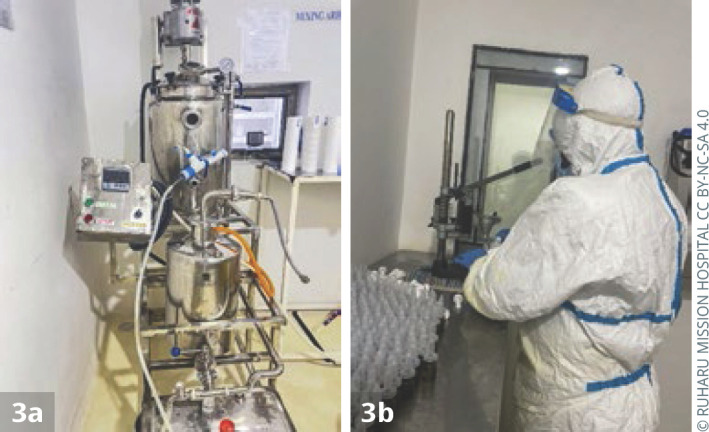
Ruharo Mission Hospital eye drop production facility **a.** Batch processing unit. **b.** Manual capping machine.

Mixing and filtration of eye drop solutions is done using a batch processing unit (Figure 3), comprising:

- **A 20 litre capacity mixing tank** with motored rotating pedals and water jacket which can be heated or cooled depending on required temperatures for mixing.
- **Filtration chamber with 5** **micron filter and suction pump** to provide pressure for flow of filtrate.
- **A 20 litre capacity holding tank** in which the filtrate is stored before dispensing into primary packaging glass bottles.

### 6. Filling eye drop solutions into bottles

- The eye drop solutions are dispensed in a specific volume into bottles using a metered ‘pressmatic’ dispenser.
- The caps and droppers (pipettes) are then press-gripped onto the bottles using a manual capping machine (Figure 3).

### 7. Sterilisation

**Figure 4:**
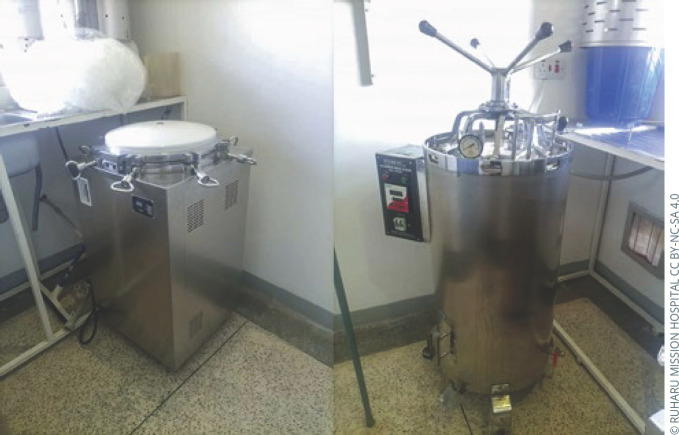
Automated autoclaves used in Ruharo Mission Hospital eye drop production facility.

Capped eye drop bottles are placed onto autoclave trays and transferred to the autoclave machines for terminal steam sterilisation (Figure 4).

### 8. Visual examination of the filled eye drop bottles

Visual inspection for leakage, particles, and clarity of eye drop solutions is done with the help of white light against both white and black backgrounds.

### 9. Quality control

A random selection of eye drop bottles from each batch is subjected to quality control analysis to assess pH, sterility, and drug content. Some of these analytical processes are in-house, whilst others are subcontracted to Mbarara University.

Products which pass the quality control tests are dispatched to the hospital stores and issued to the dispensary.
